# Amelioration of Post-traumatic Osteoarthritis by Iontophoretic Liposomal Strontium Ranelate Collaborated with Low-Intensity Pulsed Ultrasound in Rats

**DOI:** 10.3390/ijms26188815

**Published:** 2025-09-10

**Authors:** Chung-Hwan Chen, Syu-Lun Lin, Shyh Ming Kuo, Jyh-Mirn Lai, Wen-Ling Shih, Po-Chih Shen, Yi-Wen Kuo, Han Hsiang Huang

**Affiliations:** 1 Orthopedic Research Center, Kaohsiung Medical University, Kaohsiung City 80708, Taiwan; hwan@kmu.edu.tw (C.-H.C.);; 2 Regenerative Medicine and Cell Therapy Research Center, Kaohsiung Medical University, Kaohsiung City 80708, Taiwan; 3 Department of Orthopedics, Kaohsiung Medical University Hospital, Kaohsiung Medical University, Kaohsiung City 80708, Taiwan; 4 Ph.D. Program in Biomedical Engineering, College of Medicine, Kaohsiung Medical University, Kaohsiung City 80708, Taiwan; 5 School of Medicine, College of Medicine, Kaohsiung Medical University, Kaohsiung City 80708, Taiwan; 6 Institute of Medical Science and Technology, National Sun Yat-sen University, Kaohsiung City 804201, Taiwan; 7 Graduate Institute of Materials Engineering, College of Engineering, National Pingtung University of Science and Technology, Pingtung 91201, Taiwan; 8 Department of Veterinary Medicine, National Chiayi University, Chiayi City 60054, Taiwan; 9 Department of Biomedical Engineering, I-Shou University, Kaohsiung City 82445, Taiwan; 10 Department of Biological Science and Technology, National Pingtung University of Science and Technology, Pingtung 91201, Taiwan; wlshih@mail.npust.edu.tw

**Keywords:** posttraumatic osteoarthritis, liposome-encapsulated strontium ranelate, low-intensity pulsed ultrasound

## Abstract

Osteoarthritis (OA), the most common form of arthritis, affects the whole synovial joint. Post-traumatic osteoarthritis (PTOA) is an important subtype of OA which develops after joint injury. The anti-PTOA effects of iontophoretic liposome-encapsulated strontium ranelate (L-SR) combined with low-intensity pulsed ultrasound (LIPUS) were examined by a culture of human OA chondrocytes (HOACs) in alginate beads and verified on an anterior cruciate ligament transection PTOA rat model. The aim of this study is to evaluate and establish an anti-PTOA therapy combined with L-SR, transdermal iontophoresis, and LIPUS. Treatment with 10^−4^ M L-SR with LIPUS-enhanced type II collagen and glycosaminoglycans (GAGs) as L-SR with LIPUS reduced the MMP-13, IL-1β, and TNF-α in HOACs. Iontophoretic L-SR at 15 mg with LIPUS increased the weight bearing, exercise endurance, GAG density, and type II collagen intensity, while L-SR with or without LIPUS further decreased MMP13 and proinflammatory cytokines in vivo. The RBC, WBC, and serum biochemistry values were not significantly affected by the treatments. Liposome encapsulation and iontophoresis reinforce the anti-PTOA effects of SR and the addictive LIPUS further improves weight-bearing and endurance performance in the rats with PTOA. Thus, iontophoretic L-SR with LIPUS could be a potential therapy for PTOA.

## 1. Introduction

Osteoarthritis (OA) is the most prevalent form of arthritis, affecting the entire synovial joint and leading to the first cause of disability because of pain in humans [1]. OA is a complicated disease affecting the whole joint, including articular cartilage, subchondral bone, synovial membrane, menisci, tendons, ligaments, and the infrapatellar fat pad [2,3]. Post-traumatic osteoarthritis (PTOA) was caused by joint injury including ligament injury, intra-articular fracture, or other cartilage injury, accounting for about 12% of all symptomatic OA [4]. Five main risk factors have been shown to contribute to PTOA: anterior cruciate ligament (ACL) injury, meniscus tear, glenohumeral instability, patellar dislocation, and ankle instability [5], while ACL injury particularly highly occurs in adolescents playing sports. An investigation found that the incidence of PTOA reaches 87% after ACL injury [6]. Notably, the synovial membrane, as well as its functional and anatomical linking infrapatellar fat pad, play a crucial role in OA/PTOA development and progression [7,8,9]. For PTOA, it has been suggested that therapies targeted toward different pathogenic pathways may be crucial to the management of the disorder, as immediate intervention after injury is also essential to prevent future degradation [10]. Thus, early and combined anti-cytokine, anti-resorptive, and synergistic therapies may be necessary and effective to reduce the degradation of articular cartilage ECM in PTOA [11,12]. The agents with anti-resorptive effects, such as strontium ranelate (SR), bisphosphonates, and the parathyroid hormone, have also been speculated to have a disease-modifying effect in OA [13]. SR has been shown as an anti-resorptive and bone pro-forming agent to possess actions on dually and, respectively, modifying bone formation and bone resorption. The bone formation-increasing and bone resorption-reducing anti-osteoporotic dual actions of SR have been verified [14,15,16,17]. SR has therefore been shown to prevent fractures in patients with severe osteoporosis [18]. Reports showed that oral administration with SR at doses higher than the recommended dose for treating osteoporosis possesses anti-OA effects, as the anti-OA effects of SR have also been reported in vitro and on animal models [14,19,20,21,22,23]. Daily oral administration of 2 g of SR is recommended to treat osteoporosis and OA in human patients [24,25,26], despite the concern of 25% bioavailability and cutaneous and cardiovascular toxicities of SR [24]. It has been found that SR is able to reduce articular chondrocyte apoptosis in a murine OA model, as mRNA for IL-1β, MMP-13, and MMP-1 was reduced by SR treatment in a canine model [14,23]. However, the effects of SR on extracellular matrix (ECM) components and the protein levels of proinflammatory cytokines and MMP-13 were barely investigated in vitro or in vivo. Liposomes, with biocompatible, biodegradable, non-toxic, inert, and non-immunogenic characteristics, enable encapsulation and solubilization with both hydrophilic and hydrophobic compounds. The merits of liposomes include increasing stability via encapsulation of the drug, ameliorating pharmacokinetic effects and the therapeutic index of drugs, and decreasing the toxicity [27,28,29,30].

Therapeutic ultrasound, low-intensity pulsed ultrasound (LIPUS) in particular, is one of the applicable physical therapies that has been shown to possess anti-OA actions. LIPUS attenuates the deterioration of cartilage mainly via the suppression of MMP-13 mRNA and proteins in vivo [31,32]. Our previous findings further showed a combined therapy composed of the liposomal mTOR inhibitor and LIPUS with merits of enhanced anti-OA effects, a lower effective dose, and reduced administration frequency on the OA-prone Dunkin-Hartley guinea pig OA model [30]. Iontophoresis is a typical transdermal delivery route, which has several advantages, such as averting first-pass hepatic metabolism, the sustained release of drugs, and a self-administered approach to drug delivery [33]. It also provides safety and alleviation of pain over injectable administrations like intramuscular, intravenous, and subcutaneous injections [33]. We have previously found that the combination of liposomal encapsulation and iontophoresis offers propranolol anabolic effects on bone formation at lower doses and with longer administrative duration than its clinical applications [34], while other previous investigations also showed that the application of liposomes is promising in transdermal drug-delivery systems [35]. Because of the intricacy of etiopathogenesis and the clinical course of OA [36], a single therapy is unlikely to be efficient, and thus, practical and promising approaches should focus on coping with both symptoms and structural changes [11,12,37]. Although oral and injectable therapeutic agents are available for OA patients, studies indicate that most OA patients suffer persistent pain despite taking their prescribed treatments [38]. Therefore, in this study, the therapeutic and potential synergistic effects of iontophoretic liposome-encapsulated strontium ranelate (L-SR) combined with LIPUS on PTOA were examined and evaluated in HOACs in vitro and on a rat PTOA model induced by anterior cruciate ligament transection (ACLT) in vivo to develop and establish more efficacious pharmacological, physical, and transdermal therapies for the mitigation of symptoms and the improvement of structural changes in PTOA.

## 2. Results

### 2.1. Observations of L-SR by Transmission Electron Microscopy (TEM)

**Figure 1:**
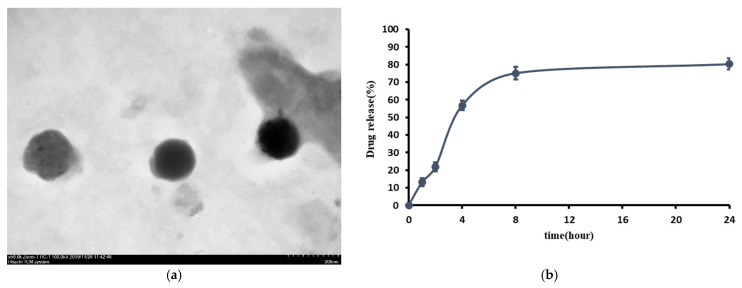
(**a**) TEM image of prepared L-SR showed an average of 126 ± 20.2 nm at magnification of 50,000×. (**b**) In vitro release profile of SR from SR-loaded liposomes (n = 3).

The TEM images of liposome-encapsulated SR fabricated in the current study are shown in Figure 1. The TEM images were captured at a magnification of 50,000×. Fairly consistent, well-dispersed, and spherical shapes of SR-loaded liposomes with an average diameter of 126 ± 20.2 nm by measuring the mean diameter of liposome were obtained (Figure 1a).

### 2.2. Encapsulation Efficiency and In Vitro Drug Release

A standard curve of SR with a sensitivity range of 10–320 μg/mL and R^2^ = 0.9997 was determined by high-performance liquid chromatography (HPLC) (Shimadzu LC-20AD prominence liquid chromatograph) (Supplementary Figure S1). The encapsulation efficiency of liposome-encapsulated SR was approximately 65.14 ± 6.3%. The in vitro release of SR from SR-loaded liposomes presented with a relatively rapid pattern within 8 h and a slow and steady drug release profile was found between 8 and 24 h. The release rate of SR from SR-loaded liposomes was about 57% from 0 to 4 h. The release from SR-loaded liposomes was around 75% after 8 h and reached approximately 80% after a 24 h incubation, respectively (Figure 1b).

### 2.3. Effects of Pure SR and L-SR in the Presence or Absence of LIPUS on HOAC Proliferation

**Figure 2:**
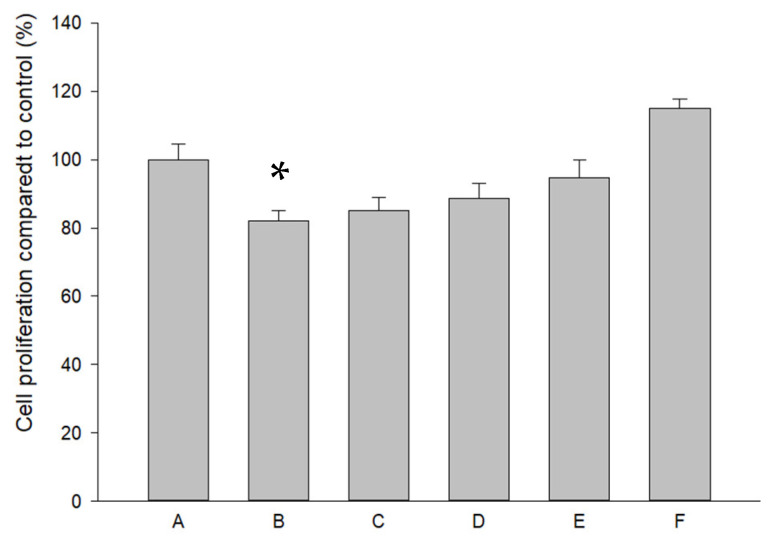
Pure SR at 10^−4^ M (Group B) significantly reduced HOAC proliferation by about 18%, and L-SR with or without LIPUS did not cause significant effects on HOAC proliferation. The letters A to F stand for the experimental groups (* *p* < 0.05; by one-way ANOVA, n = 4).

Pure SR at a high concentration (10^−4^ M) decreased HOAC proliferation (Figure 2). L-SR in the presence or absence of LIPUS did not significantly change the proliferation of HOACs cultured in alginate beads. LIPUS alone slightly but insignificantly increased DNA synthesis in HOACs (Figure 2).

### 2.4. Modulations of Pure SR and L-SR with or Without LIPUS on MMP-13, IL-1β, and TNF-α mRNA and Proteins in HOACs

**Figure 3:**
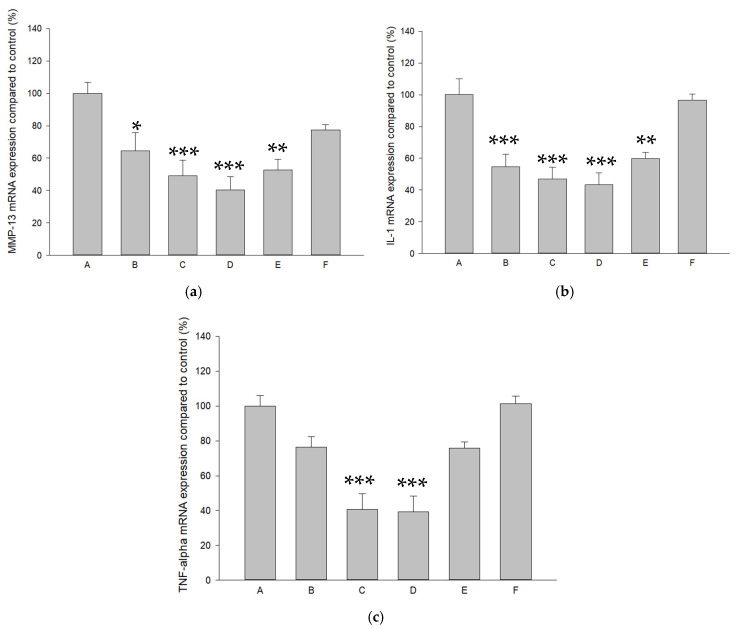
(**a**) Pure SR and L-SR in the presence or absence of LIPUS (Groups B~E) decreased MMP-13 mRNA expression in HOACs, and (**b**) mRNA for IL-1β was significantly reduced by pure SR and L-SR with or without LIPUS (Groups B~E) in HOACs. (**c**) L-SR at 10^−4^ M with or without LIPUS (Groups C and D) deceased TNF-α mRNA in HOACs (* *p* < 0.05; ** *p* < 0.01; *** *p* < 0.001 by one-way ANOVA, n = 4).

**Figure 4:**
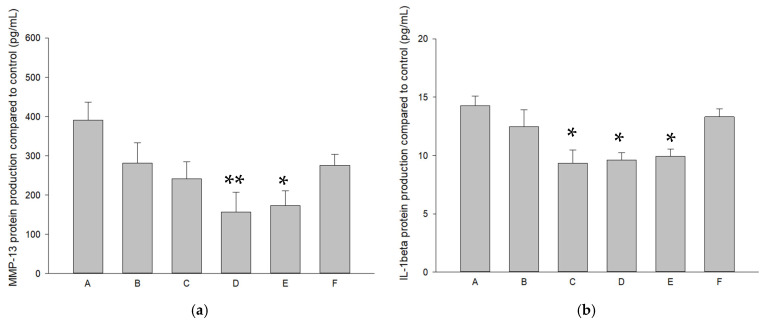

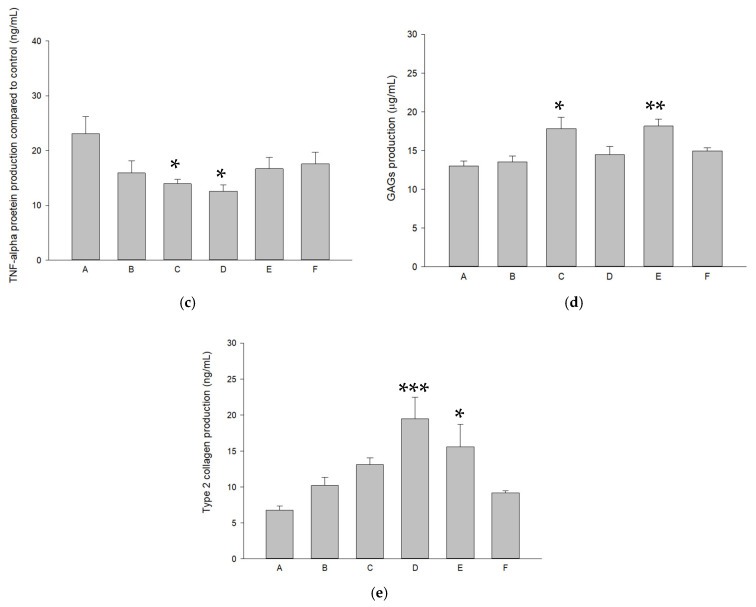
(**a**) L-SR at 10^−4^ M and 10^−5^ M together with LIPUS (Groups D and E) significantly suppressed MMP-13 protein production in HOACs. (**b**) L-SR at 10^−4^ M alone and with LIPUS and L-SR at 10^−5^ M with LIPUS (Groups C~E) inhibited IL-1β protein production, and (**c**) L-SR at 10^−4^ M in the presence or absence of LIPUS (Groups C and D) decreased TNF-α proteins produced by HOACs. (**d**) L-SR at 10^−4^ M alone and at 10^−5^ M with LIPUS (Groups C and E) increased GAG production as (**e**) 10^−4^ M and 10^−5^ M L-SR in combination with LIPUS (Groups D and E) significantly elevated type II collagen production in HOACs (* *p* < 0.05, ** *p* < 0.01, *** *p* < 0.001; by one-way ANOVA, n = 4).

The effects of pure SR and L-SR in the presence and absence of LIPUS on MMP-13, IL-1β, and TNF-α mRNA expression in HOACs were examined by real-time RT-PCR. As a result, 10^−4^ M and 10^−5^ M SR/L-SR with or without LIPUS significantly down-regulated MMP-13, IL-1β, and TNF-α mRNA expression by approximately 35–61%, 38–56%, and 22–61%, respectively (Figure 3a–c). Furthermore, L-SR at 10^−4^ M and 10^−5^ M collaborated with LIPUS significantly reduced the production of MMP-13 and L-SR at 10^−4^ M with or without LIPUS, and 10^−5^ M with LIPUS decreased IL-1β protein production by 32–40%. TNF-α protein production was also decreased by around 32–33% in HOACs in the groups of L-SR at 10^−4^ M with or without LIPUS (Figure 4a–c).

### 2.5. L-SR in Combination with LIPUS Increased GAGs and Type II Collagen in HOACs

L-SR at the high concentration (10^−4^ M) alone and at low concentration (10^−5^ M) combined with LIPUS significantly enhanced GAG production in HOACs in alginate beads (Figure 4d). On the other hand, high- and low-concentration L-SR cooperated with LIPUS significantly increased type II collagen produced by HOACs (Figure 4e).

### 2.6. Measurement of Animal Weight

**Figure 5:**
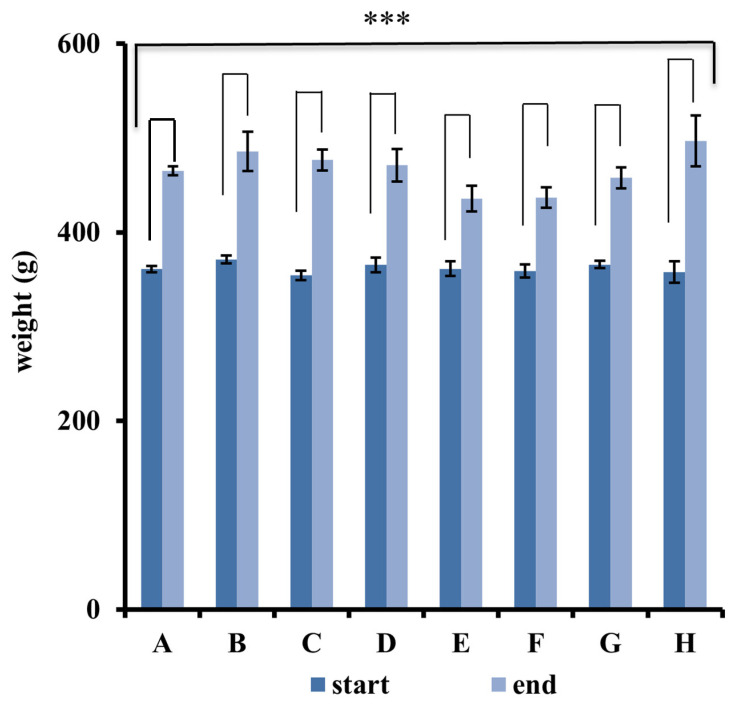
The average weights of rats in the 8 groups measured at the start and end of the experiment. Eight-group comparisons at the same time points were conducted as no significant difference was observed among experimental groups at the start or end of the experiment (by one-way ANOVA, n = 7). The comparisons in the same group at the two different time points were analyzed by Student’s *t*-test. At the end of the experiment, significant increases in average weight were found in all experimental groups compared to the respective start weight (*** *p* < 0.001; by Student’s *t*-test, n = 7).

Body weights of rats were measured at the onset and end of the experiment (Figure 5). At the start and end of 12-week experiments, no statistically significant difference in the average body weight was found among the eight experimental groups. In all study groups, significant increases in the end weight were observed compared to the initial weight (Figure 5). During the 12-week therapeutic test, no clinical signs of pain, salivation, or abnormal behavior were seen. Also, significant changes in respiratory, physical responses to stimulation, or neurological signs were not found in rats of all groups.

### 2.7. Iontophoretic L-SR Collaborated with LIPUS Improved Weight Bearing and Exercise Endurance in PTOA Rats

**Figure 6:**
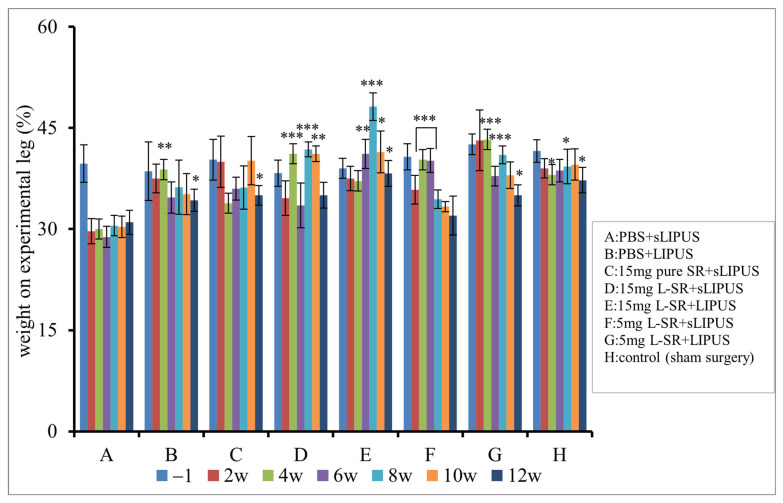
L-SR in the presence or absence of LIPUS (Groups D~G) improved the weight bearing of the affected leg in PTOA rats compared to the, respectively, identical time point of iontophoretic PBS with sLIPUS (Group A). Iontophoretic L-SR at 15 mg with LIPUS (Group E) displayed the most persistent and longest weight-bearing-improving effects on the affected leg from weeks 6 to 12. Five mg L-SR with LIPUS (Group G) possessed weight-bearing-increasing action mainly at the early and middle stages (at weeks 2, 4, and 8) as weight-bearing-increasing effects of iontophoretic L-SR at 15 mg with sLIPUS (Group D) were quite intermittent at weeks 4, 6, and 8 (* *p* < 0.05; ** *p* < 0.01; *** *p* < 0.001 by one-way ANOVA, n = 7).

Weight bearing in PTOA rats with or without the treatments is shown in Figure 6. The rats receiving sham ACLT surgery had relatively consistent weight-bearing capacities (around 40%) on the hind limb before and after the sham surgery (control, Group H). In contrast, the weight bearing of the rats in the PTOA rats receiving PBS with sLIPUS (sham LIPUS) (Group A) dropped over the 12-week experimental period. Iontophoretic 15 mg L-SR with LIPUS (Group E) ameliorated the declined weight bearing through the middle-to-late term of the experiment, exhibiting the most persistent and longest weight-bearing-improving actions on the ACLT surgery-affected leg from week 6 to week 12. Although the weight-bearing-improving effects did not occur throughout all experimental periods, iontophoretic 5 mg L-SR with LIPUS (Group G) displayed quite similar beneficial actions with those of 15 mg of L-SR with LIPUS. In contrast, iontophoretic 15 mg and 5 mg L-SR with sLIPUS showed weight-bearing-enhancing effects principally in the middle experimental periods of weeks 4 to 8 (Group D) and weeks 4 to 6 (Group F), respectively (Figure 6).

**Figure 7:**
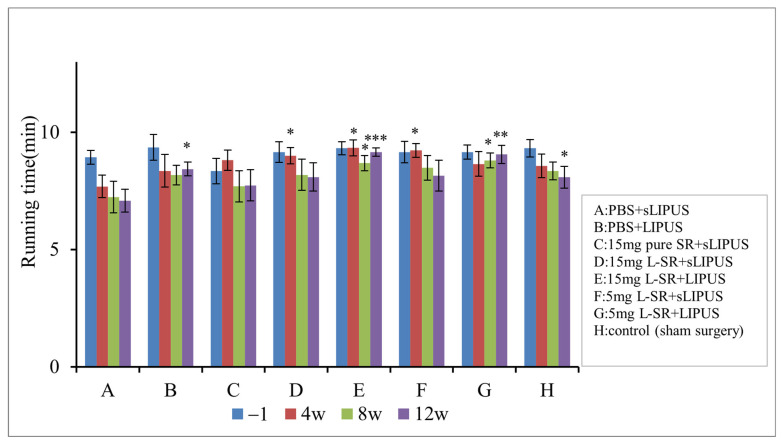
Iontophoretic 15 and 5 mg L-SR with sLIPUS (Groups D and F) increased running endurance at week 4 while 15 mg or 5 mg of L-SR in the presence of LIPUS (Groups E and G) improved the running endurance from weeks 4 to 12 compared to that at the, respectively, same time point in the rats treated with iontophoretic PBS with sLIPUS (Group A). LIPUS alone (Group B) elevated PTOA rat running endurance at week 12 compared with the, respectively, identical time point of Group A (* *p* <0.05; ** *p* < 0.01; *** *p* < 0.001 by one-way ANOVA, n = 7).

The results of running endurance are predominantly in coherence with the weight-bearing data (Figure 7). The rats in iontophoretic PBS with sLIPUS (Group A) gradually lost their exercise endurance during the 12-week experimental period. Iontophoretic 15 mg and 5 mg L-SR together with LIPUS (Groups E and G) exhibited the greatest running endurance-maintaining effects compared to those of Group A. Iontophoretic 15 mg and 5 mg L-SR with sLIPUS (Groups D and F) maintained the running endurance at the early stage of week 4 but could not be sustained to the end of the experiment. Iontophoretic PBS with LIPUS (Group B) increased the running endurance only after a 12-week treatment in the PTOA surgery rats.

### 2.8. Histology and Immunohistochemistry (IHC)

**Figure 8:**
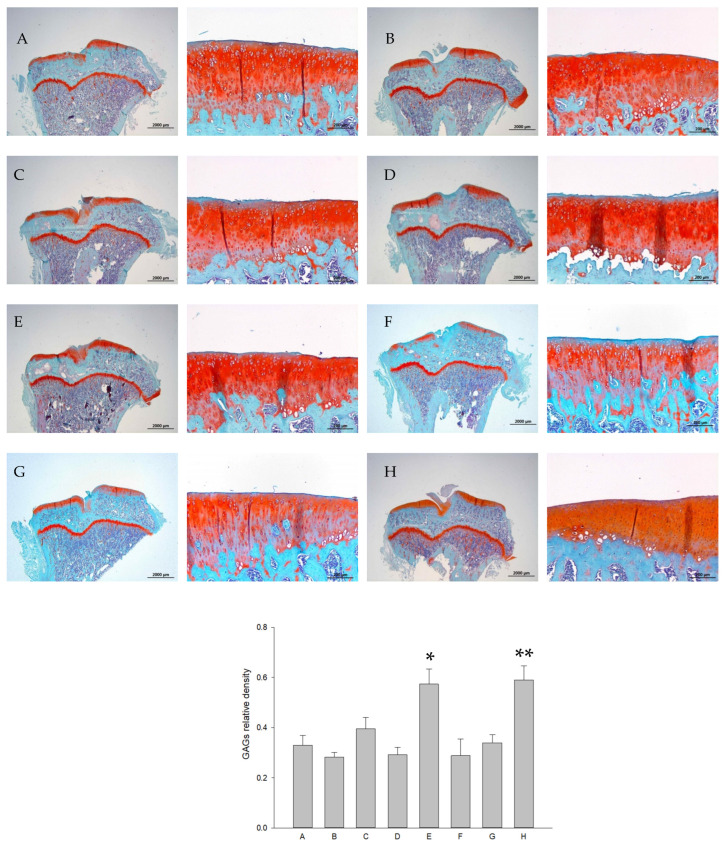
Rats that received ACLT surgery were administered with iontophoretic pure SR or L-SR with LIPUS or sLIPUS twice a week for 12 weeks. The letters A to G stand for the experimental groups. Iontophoretic 15 mg L-SR with LIPUS (Group E) significantly enhanced GAG content compared to that of iontophoretic PBS with sLIPUS (Group A). The GAG level of Group E was close to that of rats receiving sham surgery (Group H) (* *p* < 0.05; ** *p* < 0.01; by one-way ANOVA, n = 7).

The representative areas of safranin O-stained GAG intensity in the articular cartilage are shown in Figure 8. Significant increases in GAGs were seen in the group of iontophoretic 15 mg L-SR with LIPUS (Figure 8, Group E) compared to those of rats receiving iontophoretic PBS with sLIPUS (Figure 8, Group A), indicating that iontophoretic L-SR combined with LIPUS provides GAG-enhancing actions in PTOA rats in vivo. The GAG level in cartilage was close to that of the rats with sham surgery (Group H). Iontophoretic pure SR or 5 mg of L-SR with LIPUS or sLIPUS was unable to exhibit anabolic effects in the cartilage of PTOA rats (Figure 8).

**Figure 9:**
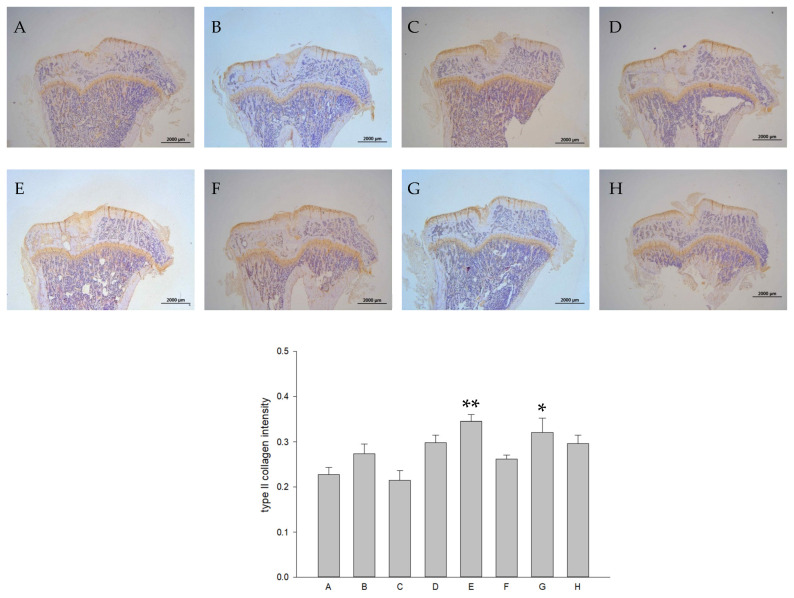
Iontophoretic L-SR at 15 mg and 5 mg, collaborated with LIPUS (Groups E and G), increased type II collagen IHC intensity in the cartilage of the knee in ACLT rats by approximately 1.6- and 1.4-folds, respectively, compared to iontophoretic PBS with sLIPUS (Group A). The IHC intensity of type II collagen was presented at 12.5 magnification. The images were quantified using Image-Pro plus 5.1 (* *p* < 0.05; ** *p* < 0.01; by one-way ANOVA, n = 7).

**Figure 10:**
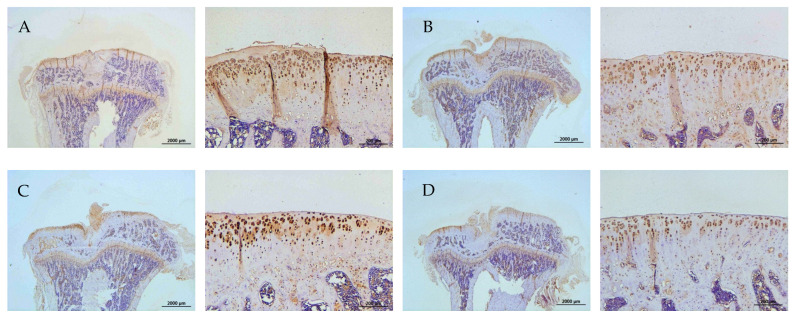

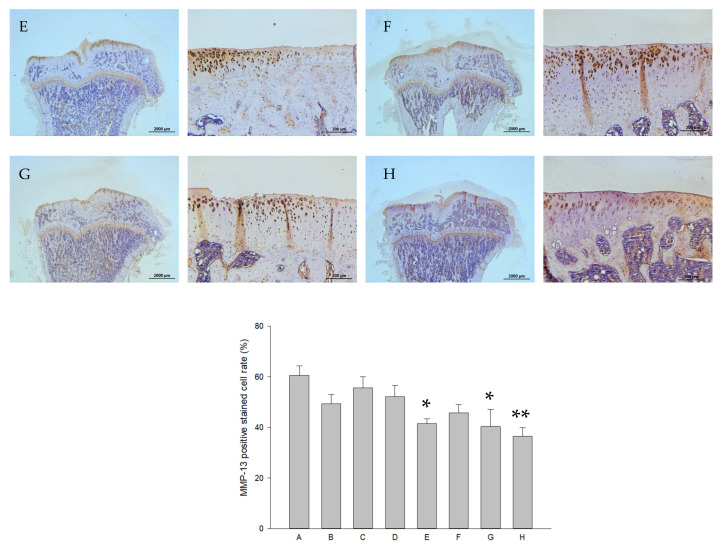
Iontophoretic L-SR at 15 mg and 5 mg with LIPUS (Groups E and G) decreased MMP-13 stained by IHC in the cartilage of PTOA knees compared to treatment with iontophoretic PBS with sLIPUS (Group A). The intensity of IHC was represented at 12.5 and 100 magnification and measured by Image-Pro plus 5.1 (* *p* < 0.05; ** *p* < 0.01; by one-way ANOVA, n = 7).

**Figure 11:**
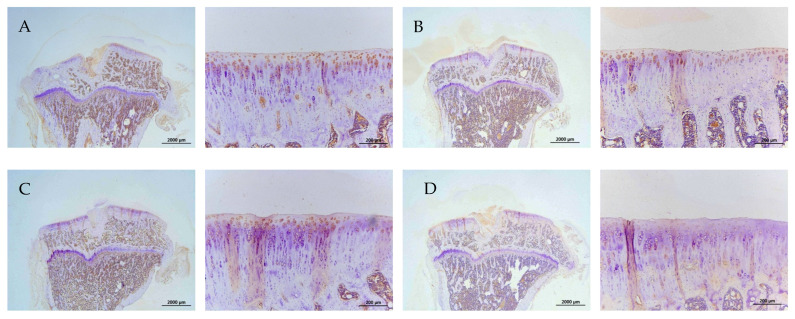

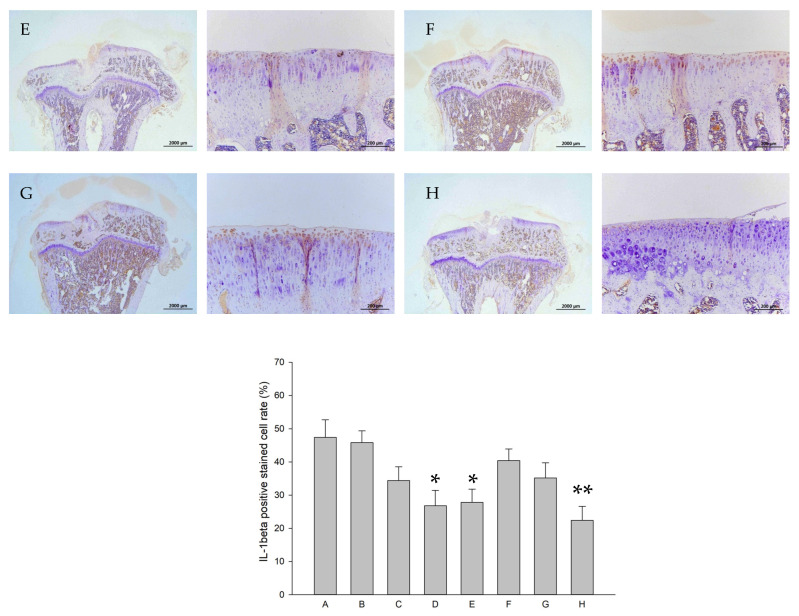
Iontophoretic 15 mg L-SR with LIPUS or sLIPUS (Groups D and E) significantly reduced IL-1β by around 42–44% stained by IHC in the cartilage of PTOA knees compared to that of iontophoretic PBS with sLIPUS (Group A) (* *p* < 0.05; ** *p* < 0.01; by one-way ANOVA, n = 7).

**Figure 12:**
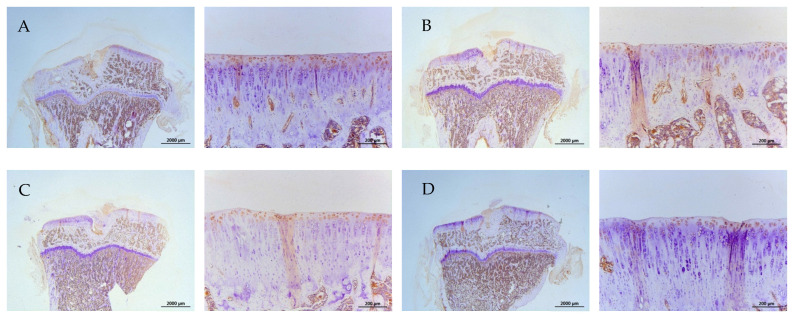

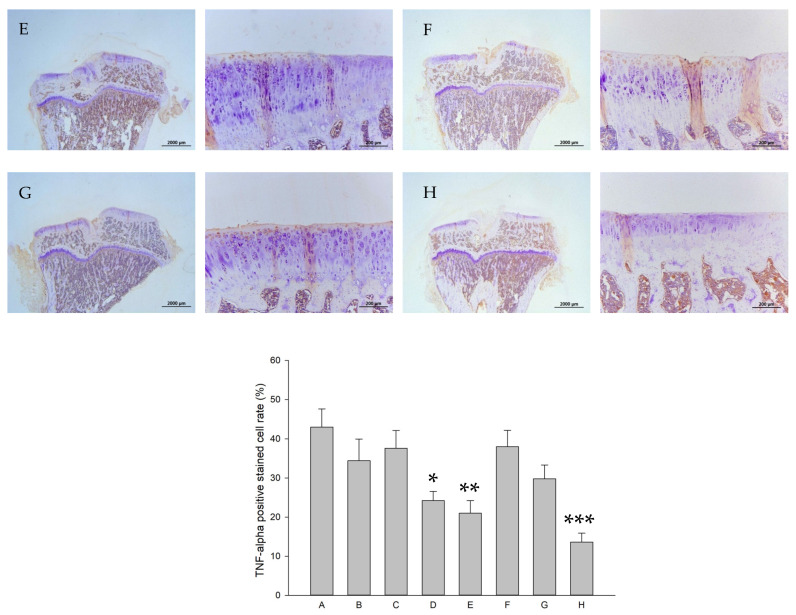
Iontophoretic 15 mg L-SR with LIPUS or sLIPUS (Groups D and E) significantly decreased TNF-α by approximately 44~50% compared to that of iontophoretic PBS with sLIPUS (Group A) in the cartilage of PTOA knees. The IHC images of IL-1β and TNF-α were represented at 12.5 and 100 magnifications, and IHC intensity was assessed by Image-Pro plus 5.1 (* *p* < 0.05; ** *p* < 0.01; *** *p* < 0.001; by one-way ANOVA, n = 7).

The representative immunohistochemical intensity of type II collagen (relative density of the brown-stained area to the total area), MMP-13, IL-1β, and TNF-α (positive brown-stained cell rate) in the articular cartilage of the PTOA knee of rats is represented in Figure 9, Figure 10, Figure 11 and Figure 12, respectively. L-SR at 15 mg and 5 mg with LIPUS significantly increased type II collagen intensity (Figure 9, Groups E and G) compared to the received iontophoretic PBS with sLIPUS (Figure 9, Group A). On the other hand, iontophoretic L-SR at both doses with LIPUS decreased MMP-13 intensity in the cartilage of the PTOA knee (Figure 10, Groups E and G) compared to that of iontophoretic PBS with sLIPUS (Figure 10, Group A). PBS with LIPUS and L-SR or pure SR with sLIPUS caused insignificant suppression of MMP-13. Fifteen mg of L-SR with or without LIPUS (Figure 11 and Figure 12, Groups D and E) significantly decreased IL-1β and TNF-α amounts in the PTOA cartilage compared to PBS with sLIPUS (Figure 11 and Figure 12, Group A).

### 2.9. Effects on RBC, WBC, Platelets, and Biochemistry

The influence of iontophoretic pure SR and L-SR with LIPUS or sLIPUS twice a week on complete cell count and serum biochemistry in rats with PTOA was assessed. WBC and RBC in all the experimental groups were recorded in the normal ranges of rats as mild increases in platelets were found in the groups of 15 mg of L-SR with sLIPUS or LIPUS [39,40,41,42] (Table 1). The values of serum biochemistry in all groups were in the normal range [39,40,41,42,43] (Table 2).

## 3. Discussion

OA is the most common type of arthritis, being the leading cause of mobility-related disability in humans, and is related to substantial individual and societal burdens [5,44]. OA is featured with its complex pathogenesis, unclear molecular mechanisms, as well as various risk factors such as aging, obesity, traumatic joint injury, inflammation, genetic factors, female sex, malalignment, and lower muscle mass [2,44,45]. In OA, chondrocytes have been shown to contribute to the inflammatory process by not only producing and releasing inflammatory cytokines and cartilage-degrading enzymes but also responding to inflammatory cytokines from other crucial sources, such as the synovial membrane and infrapatellar fat pad, resulting in aggravating inflammatory progression and cartilage degeneration [8,46]. Because of the etiologic and pathological intricacy and trickiness of OA, single therapy is unlikely to be efficient. OA principally affects the elderly, whereas sports-related traumatic injuries at all ages may result in post-traumatic OA (PTOA) [2]. In PTOA, proinflammatory cytokines such as TNF-α and IL-1β pathologically play crucial roles. These cytokines increase the level of MMPs and lead to a loss of ECM due to the activation of catabolic pathways and the inhibition of anabolic activities [47,48]. The increases in IL-1β, IL-6, and TNF-α, furthermore, have been shown to be associated with the morphological score of PTOA after ACL surgery in vivo [49]. SR has been shown to diminish the progression of OA in the cartilage and subchondral bone on animal models [14,23]. We have previously found that liposomal modification combined with LIPUS augmented the anti-OA effects of mTOR inhibitor rapamycin and lowered the effective dose and administration frequency in the Dunkin-Hartley guinea pig spontaneous OA model [50]. The route of administration of SR is mainly oral ingestion, as around 20–25% bioavailability of oral administration with SR has been reported in human patients [24]. Iontophoresis is a type of transdermal administration route to avoid or largely reduce first-pass effects. In our previous investigation, a combination of iontophoresis with liposomal encapsulation was successfully applied to the reinforcement of the anti-osteoporotic actions of propranolol [34]. Despite low bioavailability, iontophoresis together with liposomal encapsulation successfully enhanced bone formation at a lower applied dosage and with longer administrative duration in our previous work [34]. The features and size of SR-loaded liposomes were similar to our previous findings [30,51], as the capability of penetrating the skin barrier of liposome-encapsulated drugs has been verified [34]. To further avoid the influence of the first-pass effect and elevate the bioavailability of SR, liposomal encapsulation and transdermal iontophoresis were applied in the current study. Hence, potentially pharmacodynamic elongation and reinforcement of SR by liposome encapsulation in collaboration with iontophoresis and LIPUS was examined, and a cooperative therapy with clinically applicable doses and administrative frequency was established to evaluate the therapeutic effects on inflammatory, anabolic, and catabolic aspects in PTOA.

The anti-OA effects of strontium in animal chondrocytes or mesenchymal stromal cells have recently been studied in vitro. These include the promotion of chondrogenesis, the regulation of chondrocyte differentiation and proliferation, the suppression of IL-1β and MMP-13, and the elevation of aggrecan/PG and type II collagen at the mRNA and protein level [19,20,21,22]. The work by Wang et al. showed that strontium at 1 mM increases type II collagen and IGF-1 mRNA levels in rat chondrocytes [52]. Meanwhile, our previous study has shown that LIPUS alone exerted slight anabolic effects on GAGs and type II collagen production in vivo [30]. These results partially support the idea that L-SR together with LIPUS enhances type II collagen and GAGs in vitro and in vivo in the current study. On the other hand, a previous study showed that SR at 0.125–0.5 mmol/L slightly but insignificantly affected normal rat chondrocyte viability [20], whereas in the current results, SR at 10^−4^ M slightly and significantly decreased HOAC proliferation. The report performed in rat chondrocytes did not use alginate or agarose beads for the culture cells [20] and thus the maintenance of the original phenotype of chondrocytes could not be accomplished [53]. Also, although OA chondrocytes can still exert synthetic activities, the cells do not proliferate when encapsulated in scaffolding materials like alginate beads [53]. These may be the reasons why, in the current work, pure SR at 10^−4^ M slightly reduced the proliferation of HOACs in alginate beads. Our in vitro data also found that L-SR with or without LIPUS reduced MMP-13 and IL-1β at the mRNA level, while the identical treatments decreased MMP-13 and IL-1β at the protein level. These anti-MMP13 and anti-inflammatory data in HOACs are highly coherent with the strontium data in the previous in vitro studies [19,20,21,22]. On the other hand, the previous work showed that SR is able to recover type II collagen and aggrecan at the protein level under IL-1β or XAV-939 suppressive actions at higher concentrations (0.25 or 0.5 mmol/L) [19,20]. Thus, it is reasonable that our current in vitro work found that at the lower concentration (10^−4^ M), pure SR alone slightly but insignificantly increased type II collagen and GAGs (Figure 4). Our findings showed that liposomal encapsulation together with LIPUS enables SR at lower concentrations (10^−4^ M and 10^−5^ M) to significantly promote type II collagen and GAG production in HOACs in vitro (Figure 4), implicating that the combination of nanoparticle encapsulation and LIPUS is potentially essential and useful for SR with an anabolic aspect to treat OA in vivo.

The associations of a reduction in IL-1β and MMP-13 and the remodeling of the subchondral bone of SR have been implicated with its anti-PTOA actions [14,23]. Whether a reduction in the other major inflammatory cytokines, such as TNF-α, is involved in the anti-PTOA effects of SR has not been clarified. Moreover, the oral frequency of administration together with high doses of oral SR for OA therapy in the previous studies should be noted [14,23], since daily administration may result in inevitable difficulties of clinical application in humans or companion animals. In the current study, it is noteworthy that the collaboration of liposomal encapsulation, iontophoresis, and LIPUS clearly gains SR reinforcement of anti-OA actions, decreases in doses, as well as administrative frequency in vivo. In accordance with previous findings, IL-1β and MMP-13 are also associated with the iontophoretic L-SR- and LIPUS-collaborative therapy in our current work. We further found that the modulations of type II collagen, GAGs, and TNF-α are involved in the anti-OA actions exerted by the combined therapy in vitro and in vivo (Figure 4, Figure 8, Figure 9 and Figure 12). Furthermore, the benefits of liposomal encapsulation and LIPUS for potential anti-OA therapies have been found, which are that the therapeutic effects of intra-articular injection of the mTOR inhibitor on OA were strengthened [30]. In the present study, we further ascertained that iontophoresis, liposomal encapsulation, and LIPUS are capable of reinforcing the anti-OA actions of SR in vitro and in vivo. Data of type II collagen together with GAGs in the current study showed that both liposome encapsulation and LIPUS are necessary for SR to exert ECM content-promoting effects in PTOA knees (Figure 8 and Figure 9). Pure SR, L-SR, or LIPUS alone did not cause significant increases in GAGs or type II collagen, suggesting that the collaboration of liposome encapsulation with LIPUS is important for ECM-increasing effects in the cartilage of PTOA knees when a lower dose of L-SR and reduced administration frequency are administered (Figure 8 and Figure 9).

The results indicated that our treatments, composed of the iontophoretic transdermal route of SR or L-SR, liposomal encapsulation, and LIPUS, exert strong inhibitory actions on OA-correlated catabolic and inflammatory factors such as MMP-13, IL-1β, and TNF-α in vitro and in vivo (Figure 3, Figure 4, Figure 10, Figure 11 and Figure 12). The actions revealed in the current work are quite consistent with the previous in vivo anti-OA data of SR shown by the research team of Pelletier and Yu [14,23]. In contrast, moderate increases in type II collagen and GAGs were found in the groups treated with L-SR together with LIPUS in vitro and in vivo. Slight-to-moderate GAG-enhancing and MMP-13-reducing effects of LIPUS alone have been shown in our previous investigation [30]. The GAG- and type II collagen-increasing actions of L-SR with or without LIPUS in vivo are possibly the consequence of the inhibition of MMP-13, IL-1β, and TNF-α throughout the experimental period [30,31]. It is noteworthy that L-SR at higher doses in the presence or absence of LIPUS in vivo significantly decreased the IHC amount of IL-1β and TNF-α, which is consistent with that at the higher concentration with or without LIPUS, and L-SR decreased the production of the two major inflammatory cytokines in vitro. Data on the patterns of IL-1β and TNF-α reduction seem a bit different from the results of MMP-13. L-SR at low and high doses combined with LIPUS principally reduced MMP-13 production in vivo and in vitro, whereas L-SR at a high dose/concentration alone showed relatively weaker MMP-13 inhibitory actions. These results imply that L-SR itself at higher doses is capable of reducing inflammatory cytokines IL-1β and TNF-α, whereas with the assistance of LIPUS, L-SR displays clearly stronger MMP-13 inhibitory actions. Although L-SR combined with LIPUS exhibited anti-catabolic and anti-inflammatory actions that seemed stronger than its anabolic effects, the collaborative therapy increased the ECM contents to some extent. The GAGs, type II collagen, MMP-13, IL-1β, and TNF-α data further verified that the greatest anabolic and anti-catabolic effects against PTOA were exerted by iontophoretic L-SR cooperated with LIPUS.

Meanwhile, the results of weight-bearing and exercise endurance assessment also showed that iontophoretic L-SR combined with LIPUS is practically and physically effective against PTOA in vivo. Among the experimental groups, L-SR at 15 mg combined with LIPUS displayed the greatest performance in weight bearing and exercise endurance compared to that of iontophoretic PBS with sLIPUS (Figure 6 and Figure 7). It should be noted that 15 mg of L-SR combined with LIPUS also coherently represented the strongest MMP-13- and inflammatory cytokine-inhibitory as well as ECM content-increasing effects in the PTOA rats (Figure 8, Figure 9, Figure 10, Figure 11 and Figure 12). Therefore, the greatest performance displayed by the 15 mg L-SR plus LIPUS group is sensibly owed to the alleviation of inflammation and degradation in the cartilage and improvement in cartilage ECM. Notably, daily LIPUS itself has been shown to possess beneficial effects on pain relief and knee functional recovery in several previous studies [54,55,56]. It is reasonable that in the current study, LIPUS alone showed inconsistent improving actions on weight bearing, whereas it displayed significant exercise-endurance-promoting effects after long-term administration (week 12) (Figure 6 and Figure 7). In contrast, iontophoretic 15 mg L-SR with sLIPUS had mid-term weight-bearing-increasing actions at weeks 8 and 10 and early endurance-promoting effects at week 4, suggesting that iontophoretic 15 mg L-SR alone is capable of enhancing weight bearing and physical activity to some extent but is unable to maintain these actions in the absence of LIPUS collaboration. Five mg of L-SR with sLIPUS merely increased exercise endurance but could not cause significant improvement on weight bearing, whereas with the aid of LIPUS, iontophoretic 5 mg L-SR exhibited the second-best weight bearing-ameliorating and nearly identical exercise performance (Figure 6 and Figure 7). This is in accordance with our previous findings on LIPUS and further confirms that administration with LIPUS is beneficial for the recovery of knee OA, particularly as a collaborative therapy [30]. These findings further demonstrate that iontophoretic 15 mg L-SR with LIPUS may clinically exert anti-PTOA actions in vivo. On the other hand, in the current study, the sham surgery group (control, Group H) did not show higher or similar running endurance compared with that of iontophoretic 15 mg and 5 mg L-SR with LIPUS. The possible reason could be that the highest body weight was observed in the sham surgery rats among experimental groups, despite statistical insignificance, as the reduced body weight and increased exercise endurance simultaneously occurred after a designed physical activity experiment [57].

Application of iontophoresis together with liposomal encapsulation was successfully established to enhance bioavailability and reduce the clinical dose, administrative frequency, and side effects of SR in the current study. Utilization of the two therapeutic advancements for SR plus LIPUS, inflammatory and degradative cytokine-reducing, ECM content-enhancing, and weight-bearing- and exercise-endurance-improving actions was displayed in PTOA rats. Also, transdermal iontophoresis with LIPUS or sLIPUS in the current study principally did not affect RBC, WBC, and serum biochemistry in the rats, despite slight increases in platelet values found in a few iontophoretic groups. This may be due to the sedation and conduction of iontophoresis-causing stress in rats, which practically will not occur when humans execute the transdermal administration.

## 4. Materials and Methods

### 4.1. Materials

Cholesterol, octadecylamine, 1, 2-distearoyl, L-α-phosphatidylcholine (DSPC, MW: 790.15 Da), strontium ranelate, and safranin O-Fast Green were obtained from Sigma (St. Louis, MO, USA). The BrdU Cell Proliferation Kit was purchased from Merck Millipore (Burlington, MA, USA). The glycosaminoglycan assay kit was from Blyscan (Carrickfergus, UK). The type II collagen detection kit was acquired from Chondrex (Redmond, WA, USA). TRIZOL Reagent was from Invitrogen (Carlsbad, CA, USA). The reverse transcription Advantage RT-for-PCR Kit was from Takara (Kusatsu, Shiga, Japan). SYBR^®^ Green Realtime PCR Master Mix was from Bio-Rad (Hercules, CA, USA). The MMP-13, IL-1β, and TNF-α ELISA kit and horseradish peroxidase-diaminobenzidine detection immunohistochemistry kit were obtained from Abcam (Cambridge, UK).

### 4.2. Production of DSPC L-SR

The liposomes were fabricated using the evaporation sonication method with some modifications according to our previous studies [30,51]. The phospholipids were produced by a mixture of DSPC, cholesterol, and octadecylamine (OCT). Methanol chloroform (1:1, *v*/*v*) was used to dissolve the powder form of DSPC, cholesterol, and OCT, and then the liposomes were loaded into round-bottom flasks. The flask was placed in a laminar flow hood and air dried for 24 h to produce a thin layer. Nitrogen gas was then used to complete the removal of the residues of the organic solvent. Afterward, rehydration of the thin film and 20 min sonication were performed to produce L-SR [30,51].

### 4.3. Transmission Electron Microscopy (TEM)

The morphology of liposomes was analyzed by TEM. The fabricated liposome-encapsulated SR (L-SR) was placed on formvar-coated copper grids and air dried. L-SR was negatively stained with 1.5% phosphotungstic acid. Afterward, the morphology of L-SR was analyzed on a Tecnai Hitachi H7700 transmission electron microscope (Hitachi, Tokyo, Japan).

### 4.4. Encapsulation Efficiency and In Vitro Drug Release

The fabricated SR-loaded liposomes were separated from the free SR by the Sephadex G-50 minicolumn centrifugation technique, as previously shown [51]. Release of the drug from the liposomes was carried out by incubation of the filtrate with 0.25% Triton X-100 at 68 °C for 5 min. At each assigned time point, filtered SR-loaded liposomes were transferred to centrifuge tubes and refilled with an equal volume of PBS. The aliquots were filtered and diluted with a 0.25% Triton X-100 tube, followed by a 37 °C water bath at a shaking rate of 40 rpm [51]. The amount of encapsulated SR was quantified by high-performance liquid chromatography based on the previous method [58]. The release of SR from SR-loaded liposomes was calculated by the following formula: In vitro release (%) = [(total amount of SR − residue of SR)/total amount of SR] × 100% [59].

### 4.5. Cell Culture Using Alginate Beads and LIPUS Treatment In Vitro

**Figure 13:**
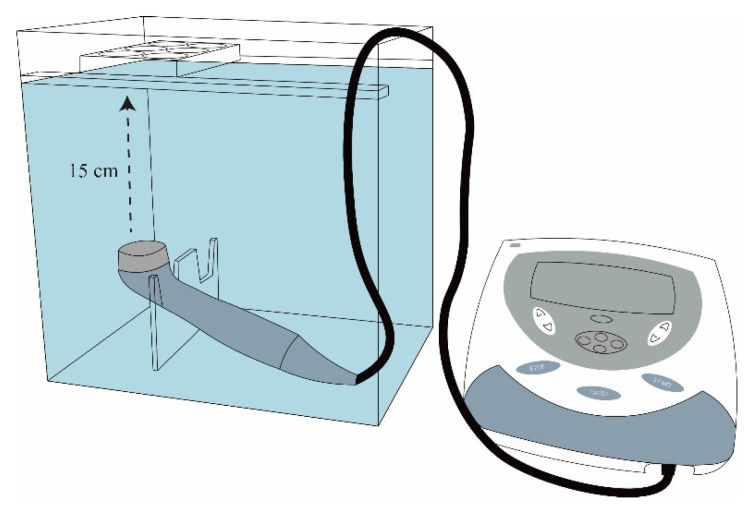
The propagation of LIPUS was through a 15 cm water layer between the ultrasonic transducer and the bottom of culture plates.

Human chondrocytes—OA (HOACs, adult, 402OA-05a) acquired from Cell Applications Inc. (San Diego, CA, USA) were primary cells from the cartilage of a 78-year-old Caucasian female OA patient. These chondrocytes were cryopreserved at the 1st passage and can be cultured and propagated to the 5th passage [30,60]. Human OA chondrocytes from the third passage were trypsinized, centrifuged, resuspended, and then cultured in alginate beads using the classical methods according to the previous reports [30,53,61,62]. HOACs at a density of 1 × 10^6^ were centrifuged, and then alginate solution (1.2% alginate) was used to resuspend the cell pellets. The cell–alginate drops were transferred to a 102 mM CaCl_2_ solution and washed with a 0.9% NaCl solution. Alginate beads were sterilely cultured in DMEM/Ham’s F-12 medium (1:1) in a humidified incubator with 5% CO_2_ at 37 °C [61,62]. LIPUS was exposed to the chondrocyte beads according to our previous study [30]. The schematic graph of LIPUS treatment is represented in Figure 13 [63]. LIPUS was generated using an Intelect Mobile Ultrasound machine (Chattanooga Group, Intelect Legend, TN, USA) designed for managing musculoskeletal, neurological, and soft tissue disorders. The cells embedded in the beads were treated with LIPUS (10 cm^2^ sound head, frequency of 1.0 MHz, duty cycle 20%, and intensity of 0.5 W/cm^2^) for 20 min/day each day for 3 days. The chondrocyte-containing alginate beads were cultured with L-SR or pure SR for 4 days with or without LIPUS treatment. The in vitro experiments were classified into 6 groups as shown in Table 3.

### 4.6. Assessment of Effects of L-SR and LIPUS on Human OA Chondrocyte Proliferation

The effects of iontophoretic L-SR in the presence or absence of LIPUS on human OA chondrocyte proliferation were assessed by BrdU assay (Millipore, Burlington, MA, USA). Sodium citrate (55 mM) was used to dissolve alginate beads, and the cell pellets were collected using Spin Fix Procedure for suspension cells recommended by the manufacturer’s instructions. In brief, the cells were incubated with a BrdU labeling solution and fixed at room temperature for 30 min, followed by a 30 min incubation with Anti-BrdU IgG (peroxidase conjugate). Plates were read using a dual wavelength of 450/550 nm on a plate reader (Bio-Rad). The GAGs, type II collagen, MMP-13, IL-1β, and TNF-α protein productions of each experimental group were normalized by the respective DNA synthesis determined by BrdU assay.

### 4.7. Quantitation of mRNA for MMP-13, IL-1β, and TNF-α

mRNA quantification of *MMP-13*, *IL-1β*, and *TNF-α* in HOACs was accomplished by real-time RT-PCR. The dissociation solution containing 55 mM of sodium citrate was used to dissolve the alginate beads. Trizol (Invitrogen) was used for RNA isolation of the cell pellet according to the manufacturer’s instructions after dissolving and gently centrifuging. The concentration of RNA was determined on a NanoDrop spectrophotometer (Wilmington, DE, USA). RNA (1 μg) was then incubated with DNase-I (Invitrogen) and Moloney murine leukemia virus reverse transcriptase with 5X reaction buffer, OligodT, dNTP, and RNase inhibitor at 42 °C for 1 h on a PCR thermal cycler (Takara) for reverse transcription. Sequences of *MMP-13*, *IL-1β*, and *TNF-α* primers for real-time RT-PCR are shown in Table 4 [30,64,65,66]. The target cDNA was quantified by SYBR^®^ Green Realtime PCR Master Mix (Bio-Rad) containing human *MMP-13*, *IL-1β*, and *TNF-α* primers on the CFX Connect Real-Time PCR Detection System (Bio-Rad). Based on our previous study, with some modifications [67], the quantitative real-time PCR was accomplished as follows: denaturation at 95 °C for 3 min, followed by 35 cycles of 95 °C for 10 s, annealing at 58–61 °C for 15 s, and elongation at 72 °C for 15 s. Relative quantification of the genes was normalized by *GAPDH* using the ΔΔCt method [68].

### 4.8. Assessment of GAGs and Type II Collagen Produced by HOACs

The GAGs synthesized by HOACs in the presence or absence of pure/liposomal SR and LIPUS was quantified by Blyscan sulfated glycosaminoglycan assay (Biocolor, Northern Ireland, UK). The alginate beads were dissolved by 55 mM of sodium citrate and centrifuged at 1500 rpm for 5 min. The supernatant was removed, followed by digestion with papain solution [30,53,61,62]. The sulfated glycosaminoglycans produced by HOACs were detected at 656 nm on a microplate reader (Bio-Rad).

Type II collagen produced by HOACs was determined by a collagen II ELISA kit (Chondrex) according to the manufacturer’s instructions. In brief, the capture antibody was first added to each well and incubated at 4 °C overnight. The samples and standards with the detection antibody were placed at room temperature for 2 h. After three washes, incubation of samples and standards with streptavidin peroxidase for 1 h was carried out, followed by color reaction with o-phenylenediamine and urea hydrogen peroxide for 30 min. The reaction was stopped by the addition of sulfuric acid. The optical density (OD) values were read at 490 nm on a microplate reader (Bio-Rad).

### 4.9. Determination of MMP-13, IL-1β, and TNF-α Proteins Produced by HOACs

Production of human MMP-13, IL-1β, and TNF-α proteins by the HOACs exposed to iontophoretic SR and L-SR with or without LIPUS was assessed by the commercial ELISA kits according to the manufacturer’s instructions. Standards and cell culture supernatants of the experimental group were incubated with human MMP13, IL-1β, and TNF-α antibodies overnight at 4 °C or 2 h at room temperature. For analyses of MMP-13, IL-1β, and TNF-α proteins, the wells were then incubated with biotinylated MMP-13, IL-1β, and TNF-α detection antibody for 1 or 3 h at room temperature after 4 washes, followed by 30–45 min incubation with an HRP-streptavidin solution. The OD values were read at 450 nm on a microplate reader (Bio-Rad).

### 4.10. Experimental Animals and Treatments

The Sprague Dawley (SD) rats used in this study were purchased from BioLASCO (Taipei, Taiwan). The rats were maintained in a temperature-controlled room (21 ± 2 °C) on a cycle of 12 h light and 12 h dark in a laboratory animal center under artificial lighting. The animal center was certified by the Association for Assessment and Accreditation of Laboratory Animal Care International (AAALAC). Rats had ad libitum access to food and water throughout the experiment. At 11 weeks of age, anterior cruciate ligament transection (ACLT) or sham surgery was performed under anesthesia by Zoletil^®^ (tiletamine/zolazepam 40 mg/kg, i.p.) + xylazine (10 mg/kg, i.p.). ACLT surgery was conducted according to our previous study [50]. Briefly, the rats were in a supine position during the surgery. The right knee stayed in flexion to access the ACL. The ACL was cut near the tibia insertion using a sharp scalpel under direct vision. To make sure that the ACL was entirely transected, the anterior drawer test was performed on the knee to see more than half of the tibial plateau [50,69]. Administration with iontophoresis of SR or L-SR and LUPUS or sLIPUS in rats (Table 3) was based on our previous studies and the work by Pelletier et al. and Hsieh et al. [23,30,34,70]. LIPUS produced by a Mobile Ultrasound machine (Intelect) was applied to the rats as follows: on–off ratio of 20%, frequency of 1 MHz, irradiation intensity of 0.1 W/cm^2^, irradiation time of 20 min, and treatment head 1 cm in diameter. Similar protocols were executed in sham LIPUS (sLIPUS) except the dose was set at 0. Fifty-six 11-week-old male SD rats were administered with iontophoretic pure/liposomal SR with LIPUS or sLIPUS for 12 weeks. LIPUS was applied to the rats twice a week in Groups B, E, F, and G.

### 4.11. Iontophoresis

The SD rats in the experimental groups were administered with pure SR or L-SR via iontophoresis according to our previous work [34]. The production of voltage and current for iontophoresis was carried out by a current generator (GS610, Yokogawa, Japan) combined with a set of transdermal patches (PF 383 and PF 384, Perimed, Järfälla, Sweden). The duration of iontophoresis for transdermal delivery of SR or L-SR was performed for 2 h per day, 2 days per week, for 12 weeks. The doses of SR or L-SR were determined based on the previous findings [23] and the potentially dose-reducing merits of liposomes [27,28,29,30]. The duration and frequency of iontophoresis as well as the location of the anode and cathode were determined based on the previous reports [34,71] and the drug release profile of liposome-encapsulated SR.

### 4.12. Assessment of Weight Bearing and Running Endurance

The effects of iontophoretic L-SR and LIPUS on weight distribution in the rat knees were determined using a dual-channel weight averager (Singa Technology, Taipei, Taiwan), which independently assesses the weight bearing of each hind paw. Briefly, the weight-bearing examinations were first conducted 1 week before ACLT surgery and every 2 weeks following until the rats were euthanized. Rats were placed in an angled Plexiglas chamber, positioned so that each hind paw stepped on a separate force plate. The force exerted by each rear limb was averaged over a 5 s period, while the mean of three 5 s readings was calculated as a data point. The percent difference in the amount of weight between the left and right feet was measured to determine the change in rear paw weight distribution [50].

For the running endurance test, the rats were adapted to run on a Columbus Instruments rodent treadmill (Columbus, OH, USA), with one training session constituting 10 min/day at a speed of 10 m/min for a week before ACLT surgery. After the adaptation session, the treadmill tests were executed at week 1 before the surgery and once every 4 weeks. All rats exercised according to a program as below. The speed was 40 m/min. The limit of the recording time of running endurance was set as 10 min, while running was terminated at the maximum duration of running endurance. A mild electric shock was set at the starting location of each lane to prevent rats from moving out of the lane. The electric shock grid was adjusted to 0.2 mA, 400 V, and 1 Hz to trigger an uncomfortable stimulation but not to physically harm the rats [50].

### 4.13. Histology and Immunohistochemistry In Vivo

The rats were euthanized by an overdose of CO_2_ after a 12-week treatment. The proximal tibiae were fixed with 10% neutral buffered formalin, and the bone samples were decalcified using 10% formic acid. Five μm sections in the coronal plane of the epiphyses were carefully prepared. Safranin O-Fast Green (1% safranin O counterstained with 0.75% hematoxylin and then 1% Fast Green) and Image-Pro Plus 5.1 software (Media Cybernetics, Rockville, MD, USA) were used to stain and quantify GAGs, respectively. The relative density of the red-stained area to the total area (density/total area) was then assessed.

The assessment of type II collagen, MMP-13, IL-1β, and TNF-α IHC in vivo in the rat cartilage stained by IHC was carried out based on our previous study [30]. H_2_O_2_ (3%) was first used to block endogenous peroxidase in tissues. The cartilage was treated with 2.5% hyaluronidase (Abcam) and 1 mg/mL of Pronase (Abcam) at 37 °C for 1 h for epitope retrieval. The samples were blocked with fetal bovine serum for 1 h prior to incubation with type II collagen (1:100 dilution; Proteintech, Rosemont, IL, USA), MMP-13 (1:500 dilution; Abcam), IL-1β (1:200 dilution; Proteintech), or TNF-α (1:200 dilution; Abcam) primary antibodies at 37 °C for 4 h. The horseradish peroxidase-diaminobenzidine detection immunohistochemistry kit (Abcam) was then added, followed by counterstaining with hematoxylin. The density/total area representing the relative density of the brown-stained area to the total area was measured for the quantification of type II collagen by Image-Pro Plus 5.1 software. The immunostaining positive cells normalized with total cells (positive stain cell rate) was calculated for the quantitation of MMP-13, IL-1β, and TNF-α using Image-Pro Plus 5.1 software.

### 4.14. Examination of WBC, RBC, Platelets, and Serum Biochemistry

At the end of the experiment, the blood was collected for WBC, RBC, platelet, and serum biochemical analysis. Following this, rats were euthanized by an overdose of CO_2_. WBC, RBC, platelets, and serum biochemistry, including AST (aspartate aminotransferase), ALT (alanine aminotransferase), BUN (blood urea nitrogen), creatinine, and electrolytes sodium, potassium, calcium, inorganic phosphorus, and chloride were analyzed by Union Clinical Laboratories (UCL, Taipei, Taiwan).

### 4.15. Statistical Analysis

The in vitro test was repeated at least three times, and data were pooled from the repeated experiments. Results are expressed as means ± standard error of mean (SEM). One-way analysis of variance (one-way ANOVA) followed by the Tukey–Kramer multiple comparisons test or Student’s *t*-test was completed on GraphPad InStat 3 Software (San Diego, CA, USA) to determine whether significant differences (*p* < 0.05) existed between the experimental groups.

## 5. Conclusions

The present data indicates that the combined utilization of the transdermal route, nanoparticle encapsulation, and physical therapy is helpful and feasible to strengthen the anti-OA actions of SR. Further verification of whether the combined therapy displays equivalent anti-OA effects in humans is needed. The data in this study demonstrate that collaborative therapies can potentially be applied to managing complicated multifactorial disorders such as OA/PTOA.

## Figures and Tables

**Table 1:** The values of RBC, WBC, and platelets in the rats with ACLT after administration with iontophoretic pure SR, L-SR, or PBS with LIPUS or sLIPUS twice a week for 12 weeks or with sham surgery. No significant differences were found among the experimental groups (by one-way ANOVA, n = 7).

	RBC (10^6^/μL)	WBC (10^3^/μL)	Platelet (10^3^/μL)
control (sham surgery)	8.14 ± 0.27	6743.71 ± 729.34	809.00 ± 90.40
PBS + sLIPUS	8.28 ± 0.12	7818.50 ± 747.73	911.17 ± 10.41
PBS + LIPUS	8.15 ± 1.50	7834.20 ± 1578.17	969.80 ± 184.21
15mg pure SR + sLIPUS	8.13 ± 0.12	6190.00 ± 281.08	862.71 ± 149.00
15mg L-SR + sLIPUS	8.18 ± 0.15	6953.33 ± 262.13	1061.00 ± 63.76
15mg L-SR + LIPUS	8.76 ± 0.14	6157.71 ± 645.26	1066.57 ± 58.16
5mg L-SR + sLIPUS	8.03 ± 0.19	6655.00 ± 480.26	895.33 ± 190.05
5mg L-SR + LIPUS	8.30 ± 0.29	6150.40 ± 1328.68	883.40 ± 255.29

SR: strontium ranelate; L-SR: liposome-encapsulated strontium ranelate; sLIPUS: sham low-intensity pulsed ultrasound; LIPUS: low-intensity pulsed ultrasound; PBS: phosphate-buffered saline; RBCs: red blood cells; WBCs: white blood cells.

**Table 2:** The values of serum biochemistry in the rats with ACLT after administration with iontophoretic pure SR, L-SR, or PBS with LIPUS or sLIPUS twice a week for 12 weeks or with sham surgery. No significant differences were found among the experimental groups (by one-way ANOVA, n = 7).

	AST (U/L)	ALT (U/L)	BUN (mg/dL)	Creatinine (mg/dL)	
control (sham surgery)	167.14 ± 22.04	65.14 ± 10.66	16.99 ± 0.81	0.36 ± 0.02	
PBS + sLIPUS	134.17 ± 9.30	51.83 ± 2.75	18.20 ± 0.69	0.35 ± 0.02	
PBS + LIPUS	179 ± 64.69	61.83 ± 8.19	18.78 ± 0.42	0.39 ± 0.01	
15mg pure SR + sLIPUS	139.86 ± 6.17	59.71 ± 6.14	17.83 ± 0.46	0.36 ± 0.01	
15mg L-SR + sLIPUS	145.83 ± 13.09	87.83 ± 3.28	17.82 ± 0.71	0.37 ± 0.03	
15mg L-SR + LIPUS	150.57 ± 16.25	62.14 ± 5.63	16.40 ± 0.60	0.38 ± 0.03	
5mg L-SR + sLIPUS	131.33 ± 5.19	52.00 ± 1.88	17.52 ± 0.70	0.36 ± 0.02	
5mg L-SR + LIPUS	136.50 ± 23.45	61.83 ± 4.41	16.55 ± 0.71	0.39 ± 0.02	
	**Na (mg/dL)**	**K (mg/dL)**	**Cl (mg/dL)**	**Ca (mg/dL)**	**P (mg/dL)**
control (sham surgery)	139.93 ± 0.30	5.80 ± 0.18	99.07 ± 0.46	9.93 ± 0.08	6.56 ± 0.32
PBS + sLIPUS	140.78 ± 0.82	6.15 ± 0.25	99.75 ± 0.69	9.98 ± 0.14	6.40 ± 0.28
PBS + LIPUS	140.58 ± 0.56	6.45 ± 0.45	99.12 ± 0.33	9.88 ± 0.11	7.52 ± 0.53
15mg pure SR + sLIPUS	140.69 ± 0.69	6.06 ± 0.11	99.93 ± 0.58	9.90 ± 0.08	7.16 ± 0.18
15mg L-SR + sLIPUS	140.87 ± 0.98	6.13 ± 0.12	98.78 ± 0.90	9.93 ± 0.10	7.65 ± 0.73
15mg L-SR + LIPUS	140.97 ± 0.99	6.17 ± 0.17	99.96 ± 1.02	10.00 ± 0.11	7.37 ± 0.38
5mg L-SR + sLIPUS	141.90 ± 0.68	5.67 ± 0.47	100.72 ± 0.49	10.00 ± 0.06	6.97 ± 0.22
5mg L-SR + LIPUS	140.67 ± 0.88	6.34 ± 0.38	99.02 ± 0.74	9.97 ± 0.18	7.03 ± 0.42

AST: aspartate transaminase; ALT: alanine transaminase; BUN: blood urea nitrogen; Na: sodium; K: potassium; Cl: chloride; Ca: calcium; P: phosphorus.

**Table 3:** Illustrations of the in vitro and in vivo experimental groups.

Groups	Treatments (In Vitro)	Treatments (In Vivo)
**A**	control	Iontophoretic PBS + sLIPUS (ACLT)
**B**	10^−4^ M pure SR	Iontophoretic PBS + LIPUS (ACLT)
**C**	10^−4^ M L-SR	Iontophoretic 15 mg pure SR + sLIPUS (ACLT)
**D**	10^−4^ M L-SR + LIPUS	Iontophoretic 15 mg L-SR +sLIPUS (ACLT)
**E**	10^−5^ M L-SR + LIPUS	Iontophoretic 15 mg L-SR + LIPUS (ACLT)
**F**	LIPUS alone	Iontophoretic 5 mg L-SR + sLIPUS (ACLT)
**G**	-	Iontophoretic 5 mg L-SR + LIPUS (ACLT)
**H**	-	sham surgery (control)

SR: strontium ranelate; L-SR: liposome-encapsulated strontium ranelate; sLIPUS: sham low-intensity pulsed ultrasound; LIPUS: low-intensity pulsed ultrasound; PBS: phosphate-buffered saline; ACLT: anterior cruciate ligament transection.

**Table 4:** The sequences of real-time PCR primers.

	Forward (5′-3′)	Reverse (5′-3′)
*MMP-13*	CTTCCCAACCGTATTGATGCT	CTGGTTTCCTGAGAACAGGAG
*IL-1β*	ATGATGGCTTATTACAGTGCAA	GTCGGAGATTCGTAGCTGGA
*TNF-α*	CCCAGGGACTCTCTCTAATC	ATGGGCTACAGGCTTGTCACT
*GAPDH*	TGTTGCCATCAATGACCCCTT	CTCCACGACGTACTCAGCG

*MMP-13*: matrix metallopeptidase-13; *IL-1β*: interleukin-1β; *TNF-α*: tumor necrosis factor-α; *GAPDH*: glyceraldehyde-3-phosphate dehydrogenase.

## Data Availability

The data presented in this study are available in the paper.

## Supplementary Materials

The following supporting information can be downloaded at: https://www.mdpi.com/article/10.3390/ijms26188815/s1.
