# Supplementary figures and images for: Amelioration of Post-traumatic Osteoarthritis by Iontophoretic Liposomal Strontium Ranelate Collaborated with Low-Intensity Pulsed Ultrasound in Rats
Source: Int J Mol Sci. 2025 Sep 10;26(18):8815. doi: 10.3390/ijms26188815 (PMC12470037; doi:10.3390/ijms26188815)

## standard curve

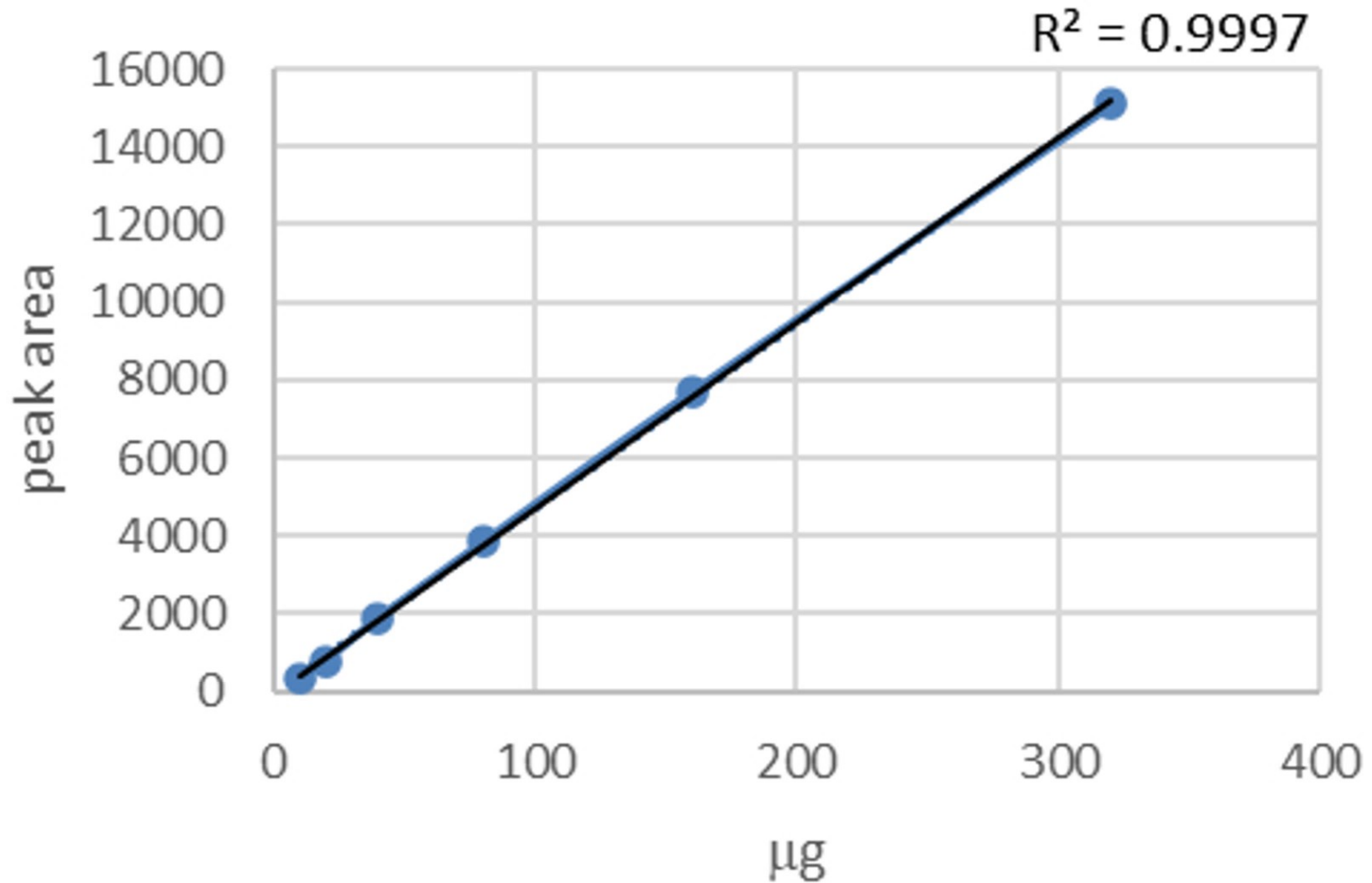

Supplementary 1. Standard curve of SR with sensitivity range of 10-320  $\mu\text{g/mL}$  ( $R^2 = 0.9997$ ).

Supplement: Supplementary file 1 [file ijms-26-08815-s001.zip › ijms-3812979-supplementary.pdf]
